# Monitoring Cattle Ruminating Behavior Based on an Improved Keypoint Detection Model

**DOI:** 10.3390/ani14121791

**Published:** 2024-06-14

**Authors:** Jinxing Li, Yanhong Liu, Wenxin Zheng, Xinwen Chen, Yabin Ma, Leifeng Guo

**Affiliations:** 1 College of Computer and Information Engineering, Xinjiang Agricultural University, Urumqi 830052, China; lijinxing1129z@163.com (J.L.); lzyfhf@gmail.com (Y.L.); 2 Agricultural Information Institute, Chinese Academy of Agricultural Sciences, Beijing 100080, China; 3 Xinjiang Agricultural Informatization Engineering Technology Research Center, Urumqi 830052, China; 4 Ministry of Education Engineering Research Centre for Intelligent Agriculture, Urumqi 830052, China; 5 Institute of Animal Husbandry Quality Standards, Xinjiang Academy of Animal Science, Urumqi 830011, China; zwx2020@126.com (W.Z.); chxw106@163.com (X.C.); 6 Hebei Animal Husbandry and Breeding Work Station, Shijiazhuang 050049, China

**Keywords:** cattle, improved YOLOv8-pose, rumination behavior, peak detection

## Abstract

**Simple Summary:**

Rumination behavior is a crucial indicator of cattle health and welfare. The timely monitoring and analysis of this behavior can provide valuable insights into the physiological status of the animals. Distinguishing from manual observation and wearable devices, this study proposes a method using video technology for monitoring cattle rumination behavior. This method aims to track physiological indicators during rumination, including chewing count, rumination duration, and chewing frequency. This approach can help livestock managers promptly understand the health status of cattle. Furthermore, this research method offers a new perspective for the construction of smart farming, providing technical support for the intelligent transformation of the livestock industry.

**Abstract:**

Cattle rumination behavior is strongly correlated with its health. Current methods often rely on manual observation or wearable devices to monitor ruminating behavior. However, the manual monitoring of cattle rumination is labor-intensive, and wearable devices often harm animals. Therefore, this study proposes a non-contact method for monitoring cattle rumination behavior, utilizing an improved YOLOv8-pose keypoint detection algorithm combined with multi-condition threshold peak detection to automatically identify chewing counts. First, we tracked and recorded the cattle’s rumination behavior to build a dataset. Next, we used the improved model to capture keypoint information on the cattle. By constructing the rumination motion curve from the keypoint information and applying multi-condition threshold peak detection, we counted the chewing instances. Finally, we designed a comprehensive cattle rumination detection framework to track various rumination indicators, including chewing counts, rumination duration, and chewing frequency. In keypoint detection, our modified YOLOv8-pose achieved a 96% mAP, an improvement of 2.8%, with precision and recall increasing by 4.5% and 4.2%, enabling the more accurate capture of keypoint information. For rumination analysis, we tested ten video clips and compared the results with actual data. The experimental results showed an average chewing count error of 5.6% and a standard error of 2.23%, verifying the feasibility and effectiveness of using keypoint detection technology to analyze cattle rumination behavior. These physiological indicators of rumination behavior allow for the quicker detection of abnormalities in cattle’s rumination activities, helping managers make informed decisions. Ultimately, the proposed method not only accurately monitors cattle rumination behavior but also provides technical support for precision management in animal husbandry, promoting the development of modern livestock farming.

## 1. Introduction

In recent years, to meet the increasing demand for beef and milk, the scale of cattle farms has continuously expanded, and the farming model has gradually shifted from traditional free-range to intensive farming. This transformation has not only significantly increased the output of cattle and the quality of dairy products but also brought about management challenges. In an intensive farming environment, monitoring the health status of each cow has become more complex and crucial.

Rumination behavior is a crucial indicator in the health monitoring of cattle. As a characteristic behavior unique to ruminant animals such as cows and sheep, researchers often observe feeding and rumination activities to monitor the health of cattle. The variations in rumination time and frequency are closely associated with the health status and physiological state of cattle [1]. Rumination monitoring can be used for estrus detection [2,3], and cows typically exhibit a noticeable reduction in rumination time before calving [4]. However, ruminating behavior monitoring for cows primarily relies on manual observation, but this method incurs high labor costs and is challenging to accurately assess the health status of cows [5]. Therefore, monitoring cows’ rumination behavior is not only crucial for promptly detecting abnormal behavior and implementing corresponding treatment measures but also holds significant importance in enhancing the automation level of livestock farming.

Currently, monitoring cows’ rumination behavior is primarily conducted through contact sensor devices such as ear tags, collars, and nose rings. These devices typically include features like positioning, pressure sensing, acceleration, and sound detection. The pressure sensors are mainly worn near the cow’s nose and mouth, directly sensing pressure changes in the jaw movement. By analyzing these changes, rumination behavior can be monitored effectively. Chen et al. [6] use a noseband pressure sensor to capture pressure changes in cows’ lower movements, using the extreme gradient boosting (XGBoost) algorithm to classify features of rumination and feeding behaviors. Zehner et al. [7] developed and validated a novel RumiWatch noseband pressure sensor, designed for automated measurement of rumination and feeding behaviors in cows. Kröger et al. [8] installed a noseband sensor system (NSS; “RumiWatch”, Itin+Hoch GmbH, Liestal, Switzerland) on the noses of cows to monitor rumination activity, replacing time-consuming manual observations of total rumination time to assess the growth of cows under different feeding conditions. Pahl et al. [9] implemented an automatic detection device using pressure sensors to analyze the impact of calving on rumination behavior in dairy cows. However, it is challenging to maintain the same initial pressure while wearing pressure sensors, which poses a certain challenge in data processing and can affect the accuracy of the results to some extent.

The acceleration sensor is primarily fixed on the head, jaw, ears, and neck of the cow. It identifies rumination behavior and other activities by analyzing the differences in acceleration under various states. Benaissa et al. [10] attached a three-axis acceleration sensor to the neck of a cow and accurately identified rumination behavior by using a new simple decision tree (DT) algorithm. Rayas-Amor et al. [11] designed a rumination behavior monitoring device based on a three-axis acceleration sensor, which was installed at the jaw position of cows to differentiate rumination behavior from other behaviors. Martiskainen et al. [12] utilized the support vector machine (SVM) algorithm to process data from acceleration sensors on the neck of cows, categorizing their behaviors into multiple classes, including feeding and rumination. Jaeger et al. [13] used accelerometer ear tags to collect behavioral activity features of cows, establishing a close connection between sensor behavior features and subjectively monitored cow behaviors, and found that high-yielding cows exhibited more intense feeding and rumination behaviors. In real-world scenarios, acceleration sensors are often influenced by similar acceleration signals, and different behaviors may have similar signal characteristics, making it difficult to distinguish behaviors [14]. Additionally, wearing sensor devices can easily trigger stress responses in cows, and the devices are prone to wear and tear, leading to high costs when applied to large-scale farming operations.

Sound sensors are generally placed at the temporal fossa and throat of cattle to analyze the sounds of chewing and swallowing, which helps distinguish the rumination behavior of cattle. Chelotti et al. [15] introduced an online bottom-up foraging activity recognizer (BUFAR) that identifies jaw movements from sounds, evaluated and compared through the following two variants: a multi-layer perceptron (BUFAR-MLP) and a decision tree (BUFAR-DT). This approach demonstrated superior performance in estimating foraging and rumination behaviors. Burfeind et al. [16] conducted experiments on dairy cows of various ages using the HR-Tag device and found that the system can accurately monitor the rumination behavior of dairy cows older than 9 months. Vanrell et al. [17] distinguished rumination from other behaviors by identifying the duration and pauses of sound signals during cattle foraging. However, in actual barn feeding or grazing conditions, audio signals are susceptible to interference from magnetic fields and noise, which can affect the accuracy of recognition.

Although contact sensor devices have high accuracy in monitoring cattle rumination behavior, they are prone to collisions and compression during long-term monitoring, which can lead to device damage and potentially raise animal welfare issues. Therefore, monitoring cow rumination behavior using non-contact computer vision methods is likely to become a future trend. Mao et al. [18] developed an automatic detection method for the oral area during cow rumination using a series of processing techniques, including grayscale conversion, achieving a maximum accuracy of 87.8% and laying the groundwork for the automatic detection of cow rumination behavior. Chen et al. [19] utilized the mean-shift algorithm to accurately track the jaw movements of targeted dairy cows by plotting the motion curve of the centroid within the mouth area images, thereby achieving the effective monitoring of cow rumination behavior. Ayadi et al. [20] used a deep learning model based on convolutional neural networks (CNN) to identify rumination behavior by monitoring all cow postures captured by cameras, achieving an accuracy rate of 98%. Wang et al. [21] employed a combination of the YOLO algorithm and the kernelized correlation filter (KCF) to count the rumination chews of cattle. The results indicated an average error rate of 8.126%. Gao et al. [22] proposed a UD-YOLOv5s algorithm, combined with mandibular skeletal feature extraction technology, to analyze the rumination chewing actions of cattle, enabling accurate identification of rumination behavior. The aforementioned studies demonstrate the effectiveness of non-contact monitoring methods, indicating that significant progress has been made in this field. In the future, non-contact computer vision methods are likely to become mainstream in research because they can effectively reduce labor costs and promote animal welfare.

At present, although research on rumination behavior based on machine vision has made some progress, several issues remain, as follows: (1) most studies are typically limited to detecting whether rumination behavior has occurred, without providing more detailed monitoring and analysis; (2) the image data processing for rumination behavior is relatively complex; and (3) the application of keypoint detection technology in the animal field is still underdeveloped, with considerable room for improvement. This study proposes a method based on an improved YOLOv8-pose keypoint detection and constructs a bovine facial keypoint dataset to verify the feasibility of the model. Subsequently, a monitoring framework for rumination behavior was established by using a multi-condition threshold peak analysis of keypoint information related to cow rumination behavior. This framework facilitates the analysis of metrics such as the number of rumination chews, rumination duration, and rumination frequency. Subsequently, a monitoring framework for rumination behavior was established using multi-condition threshold peak detection to analyze keypoint information on cow rumination behavior. This framework is designed to analyze metrics such as the number of ruminating chews, ruminating time, and chewing frequency. This method has successfully implemented the monitoring of cow rumination behavior, which lays the foundation for the development of smart farming technologies.

## 2. Materials and Methods

### 2.1. Experiments and Data Acquisition

This study initially conducted data collection on 1 August 2023 in a grassland field in Xeltala Town, Hulunbuir City, Inner Mongolia Autonomous Region, China, located at longitude 120.00435 and latitude 49.34809. Subsequently, on 18 January 2024, we continued data collection at Wangyuan Dairy in Xingtang County, Shijiazhuang City, Hebei Province, China, located at longitude 114.642334 and latitude 38.435799.

**Figure 1:**
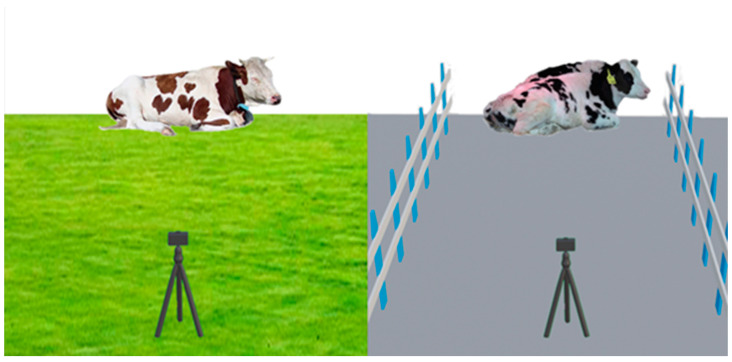
Schematic diagram of data collection scenario.

To ensure the quality and accuracy of the data, the experiment employed a handheld camera (Snapdragon S3 sports camera) for video recording. The video was set to record at 30 frames per second, with a resolution of 3840 × 2160. In the experiment, 22 cows were observed, all of which were free to move around during the entire observation period. Additionally, no harm was inflicted on the cows during the experiment. To comprehensively capture the cows’ rumination behavior, we observed them from various angles and distances. Figure 1 shows the data collection scenario; the data collection lasted for 14 days, from 8 AM to 5 PM daily. The duration of the videos varied from 10 s to 5 min, totaling 54.4 GB of video data on cow rumination.

Additionally, during the filming period, we encountered issues with the camera’s inability to continuously capture the subtle movements of the cows’ noses and mouths, especially when the cows turned their heads or were blocked by others. Additionally, due to the limitations of the experimental site and some cows’ habit of curling up in corners, our filming faced significant challenges. Therefore, we only used high-quality, complete video clips for further analysis. These selected video clips came from 12 different cows. Consequently, we decided to use only those video clips of higher quality and complete information for further analysis. These videos featured various shooting angles, including front, left, right, and rear side views, and showcased each cow’s different body shapes, colors, and postures, including standing and lying down, amounting to a total of 24.6 GB of video data.

### 2.2. Data Processing

From the original video data, we employed a frame extraction method to obtain images. This approach ensured that while no crucial information was lost, the diversity of data samples was effectively increased. In total, 2034 RGB images were extracted from the video data, with 1225 images from grazing scenes and 809 images from breeding shed scenes. These images encompass various scenarios featuring different cows in different angles, lighting conditions, and behavioral states.

**Figure 2:**
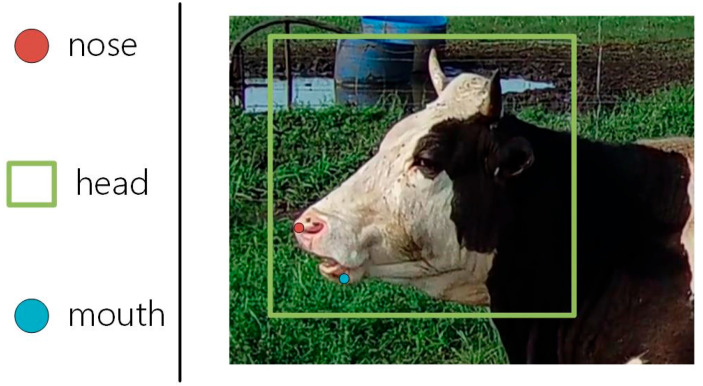
The results of the annotations.

To further capture the characteristics of cow rumination behavior accurately, we used the labelme image annotation tool to label the cow’s head and the following two keypoints: the nose and mouth. Through the precise localization of these keypoints, we can more accurately analyze and identify cow rumination behavior, laying a solid foundation for subsequent behavior analysis and research. Figure 2 shows the results of the annotations.

### 2.3. Data Enhancement

**Figure 3:**
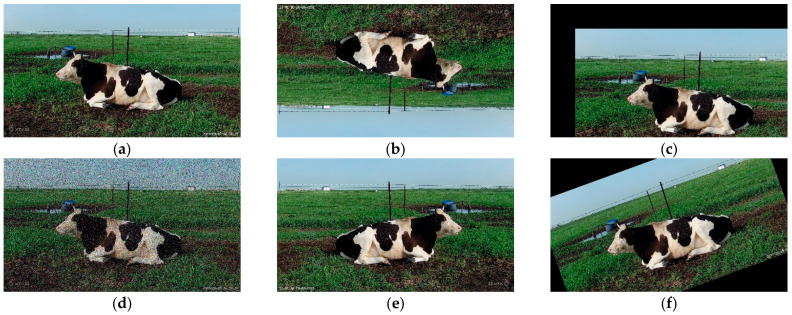
(**a**) Original image. (**b**) Image after flipping. (**c**) Image after panning. (**d**) Image after adding noise. (**e**) Image after mirroring. (**f**) Image after rotation.

To accelerate the training process of the model and promote its convergence, thereby achieving better performance and enhancing the robustness of the model, we employed data enhancement. Data enhancement involves systematically or randomly altering and expanding data through programming means. In the field of image processing, these techniques typically include flipping, translating, mirroring, and adding random noise to images [23], as shown in Figure 3. These methods effectively increase the diversity of training samples, thereby enhancing the model’s adaptability and generalization ability under different conditions.

### 2.4. Technology Line

**Figure 4:**
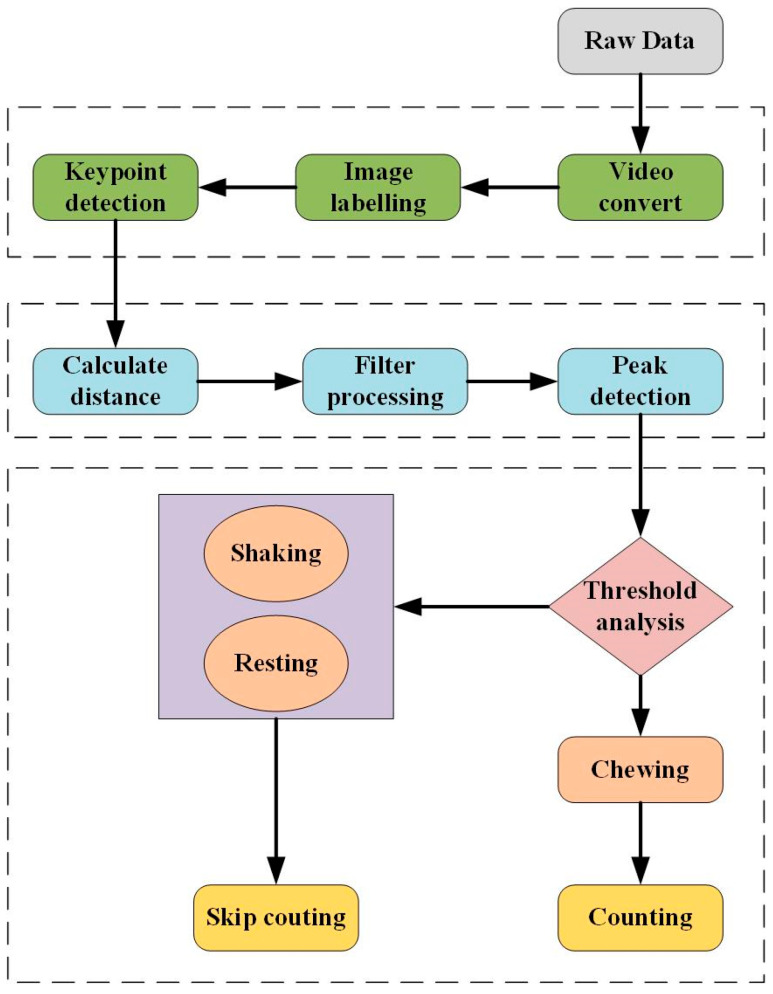
Flowchart of the research.

Figure 4 illustrates the overall technical workflow of this paper. We use an improved YOLOv8-pose model to detect keypoints on the cow’s nose and mouth. The distances of the detected keypoints are calculated and subjected to filtering. Subsequently, an appropriate threshold is applied to a peak detection algorithm to count the number of ruminating chews. Finally, the results are compared with manually observed counts to validate the feasibility of the method.

### 2.5. Model Improvement Implementation

#### 2.5.1. YOLOv8-Pose Keypoint Detection Algorithm

**Figure 5:**
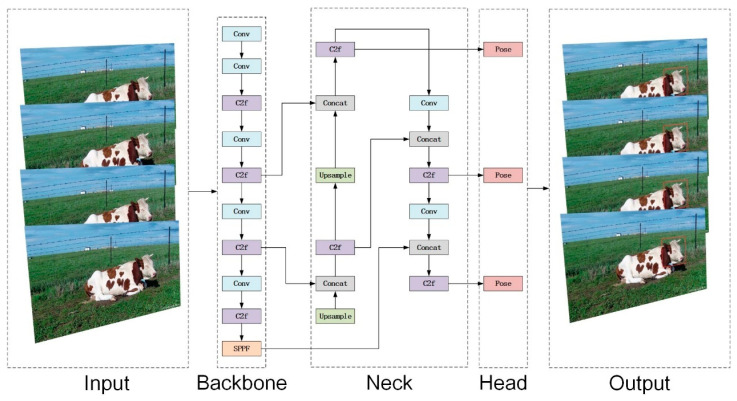
YOLOv8-pose network structure diagram.

YOLOv8-pose is a deep convolutional network based on YOLO v8, which adds keypoint detection to its target detection, primarily used for human pose estimation. Its main components include the input, backbone, neck, and head sections. The input section is responsible for resizing the input image to the dimensions necessary for training. The backbone and neck utilize the ELAN structure, enabling the C2f module within the backbone to merge and propagate features from different layers, enhancing feature expression and improving the detection performance and convergence speed of the YOLOv8-pose model. The neck features a PAN structure, bolstering the network’s ability to integrate features across varying scales, essential for merging and propagating feature information to boost model performance. The head section processes the network’s output and generates the final detection results. To better train the model and compute keypoint positions, the network employs a keypoint loss calculation module, which is used to train models capable of keypoint detection and optimizes model parameters through weighted loss. Figure 5 shows the YOLOv8-pose network structure.

#### 2.5.2. SimSPPF

**Figure 6:**
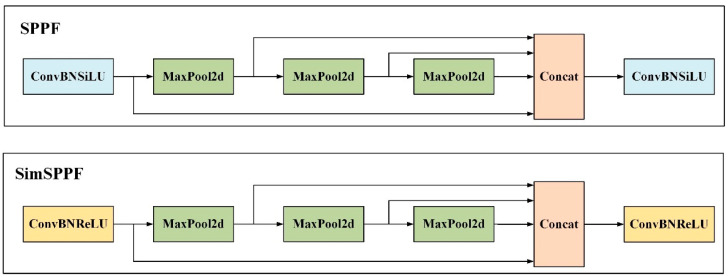
SPPF and SimSPPF structure diagram.

Simplified spatial pyramid pooling-fast (SimSPPF) is an accelerated version of spatial pyramid pooling used in object detection. It enhances the computational efficiency and speed of the target detection models by simplifying the calculation process of spatial pyramid pooling and reducing the dimensions of feature maps [24]. SimSPPF retains the multi-scale feature fusion capability of spatial pyramid pooling-fast (SPPF) while incorporating strategies for rapid computation. Specifically, it uses the ReLU activation function, enabling the model to perform better when processing large-scale data. The primary aim of this technique is to reduce computation and accelerate feature extraction while maintaining detection accuracy, thus adapting to efficient object detection applications. Figure 6 illustrates the structures of SPPF and SimSPPF.

#### 2.5.3. ECA Attention Mechanism

**Figure 7:**
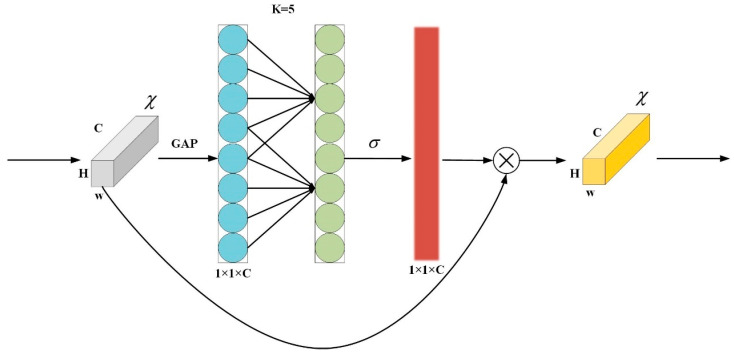
ECA structure diagram.

As an input image passes through a convolutional network, spatial information gradually transitions to channels. During this process, with each spatial compression or channel expansion of the feature image, some semantic information is lost. Therefore, channel attention mechanisms have shown great potential in enhancing the performance of deep convolutional neural networks. However, most methods aim to develop more complex attention mechanism modules to achieve better performance, significantly increasing the complexity of the model. We employ the lightweight efficient channel attention (ECA) mechanism to address the trade-off between performance and network complexity. The ECA attention mechanism is an enhancement of the SENet architecture, introducing a no-dimension-reduction local cross-channel interaction strategy [25]. This strategy is efficiently implemented through one-dimensional convolution, allowing the model to maintain performance while significantly reducing complexity, as shown in Figure 7. Specifically, the ECA attention mechanism first applies global average pooling to the input feature maps, reducing their dimensions to 1 × 1 × C. It then processes the resulting feature vector with a sigmoid activation function. Subsequently, one-dimensional convolution is used to weight the inputs, capturing information from the input image. Finally, it outputs features that incorporate channel attention.

#### 2.5.4. RepGPFN Network

**Figure 8:**
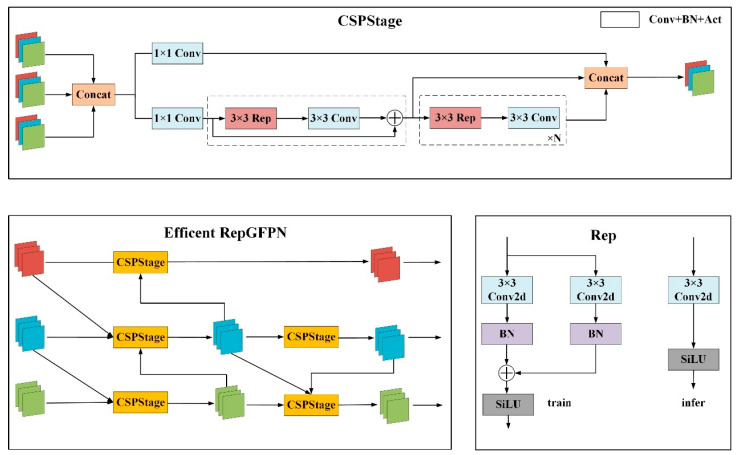
RepGFPN network diagram.

The neck module is designed to perform the deeper processing and fusion of features from the backbone network, enhancing the performance and accuracy of detection tasks, particularly in handling features of various scales. To enhance the model’s comprehension of image details and structures and capture image scale changes and complexities more comprehensively, this study introduces a re-parameterized version of the efficient re-parameterized generalized feature pyramid network (RepGFPN) [26]. This network enhances model performance by optimizing the original generalized feature pyramid network’s (GFPN) topology and feature fusion methods, as shown in Figure 8. Specifically, RepGFPN introduces a CSPStage module based on efficient layer aggregation network connections, designed to merge features from the same, adjacent, and different scales. This module effectively handles features of varying scales and complexities, enhancing feature fusion and information propagation. The input to this module has three layers, which, after a Concat operation, undergo parallel processing through two 1 × 1 convolutions to adjust channel counts. The lower branch consists of N 3 × 3 RepConv convolutions and traditional 3 × 3 convolutions. Finally, the outputs of the last two branches are combined again through a Concat operation to produce the final result.

#### 2.5.5. Improved YOLOv8-Pose Model

**Figure 9:**
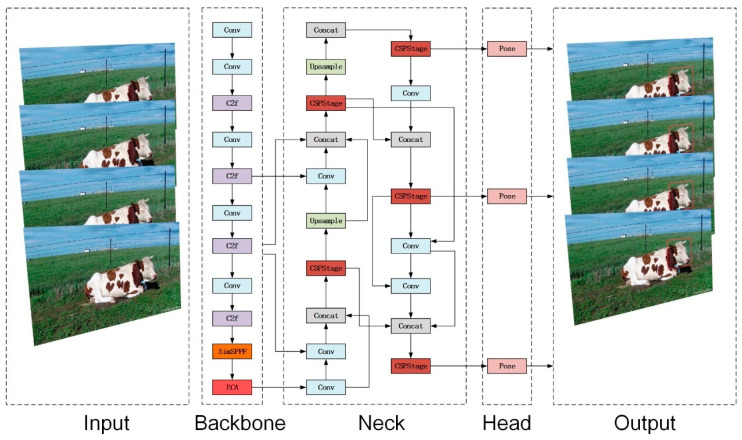
The improved network structure of the YOLOv8-pose model.

In this study, three modifications were implemented in the YOLOv8-pose model to enhance its performance. The feature extraction network was replaced with SimSPPF to reduce computational complexity and speed up model convergence. The ECA attention mechanism was introduced into the backbone feature extraction network to enhance feature representation capabilities while maintaining performance, despite the reduced complexity. In the neck, the model was reconstructed using RepGFPN to improve feature fusion and transmission capabilities, thereby optimizing overall model performance. Figure 9 shows the improved network model.

### 2.6. Ruminating Behavior Module

Using the keypoint detection model, we obtained the coordinates of the keypoints for the nose and mouth, marked as $P_{i}(x_{i},y_{i})\in \left(1,2\right)$, where *x* represents the horizontal coordinate and y represents the vertical coordinate, corresponding to the nose and mouth respectively. The variable *d* represents the Euclidean distance between the nose and mouth. The formula to calculate the distance between keypoints is shown as Equation (1).

(1) $$d=\sqrt{{\left(x1-x2\right)}^{2}+{\left(y1-y2\right)}^{2}}$$

In this study, we generated a motion curve for cow rumination based on the distance between the nose and mouth keypoints. During a single chewing cycle, the cow’s mouth movements are divided into the following three stages: mouth closing, mouth opening, and mouth closing again. As the mouth opens, the distance between the nose and mouth increases, reaching a maximum when the mouth is fully open. Once the chewing is complete and the mouth closes, the distance between the nose and mouth returns to its minimum. This creates a motion curve similar to a sinusoidal wave on the graph, characterized by reciprocal up and down fluctuations. Unlike a regular sinusoidal wave, the amplitude and frequency of each cycle vary in this motion curve because each chewing episode differs in magnitude and frequency. To count the number of chewing instances, it is necessary to tally the number of these oscillatory cycles in the motion curve. Since the frequency of these oscillations changes, it is not feasible to simply divide the total change along the horizontal axis by the frequency. Instead, each cycle must be counted individually.

**Figure 10:**
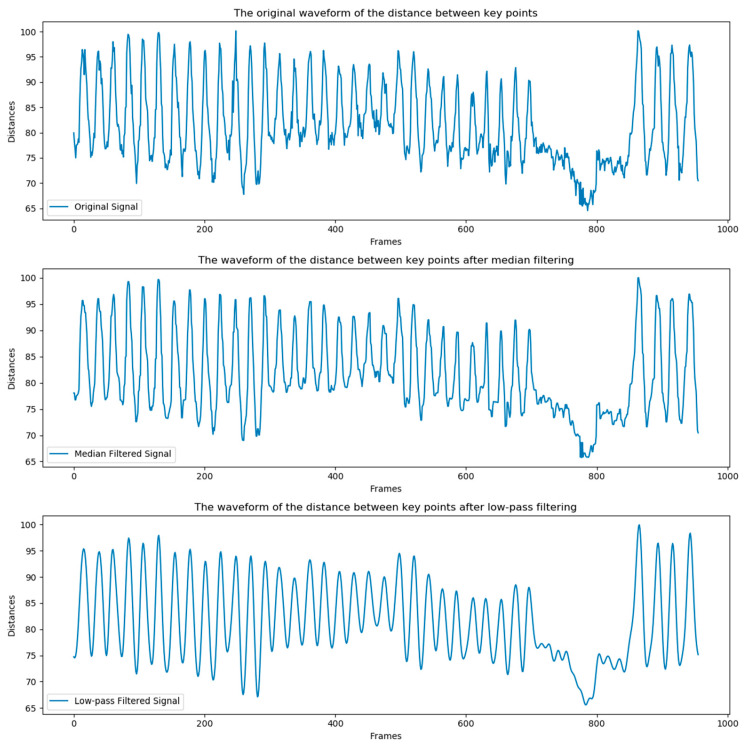
The rumination motion curve.

Counting these oscillations can be equated to counting the number of “sinusoidal wave peaks”. Ideally, each peak represents one chewing cycle. However, cows do not remain completely still during rumination, and minor jitters can cause slight deviations in keypoint detection, making the direct counting of data points in which the middle is higher than the sides inaccurate due to the unsmooth nature of the curve. Therefore, it is essential first to perform noise reduction to smooth the line on the image, making it easier to accurately count the peaks. Due to errors caused by swinging or inaccurate detection, the curve contains some high-frequency noise. Therefore, we chose a low-pass filter for noise reduction, as it can suppress high-frequency noise interference [27]. Additionally, we used a median filter for comparison and found that the low-pass filter performed better in noise reduction (See Figure 10).

**Figure 11:**
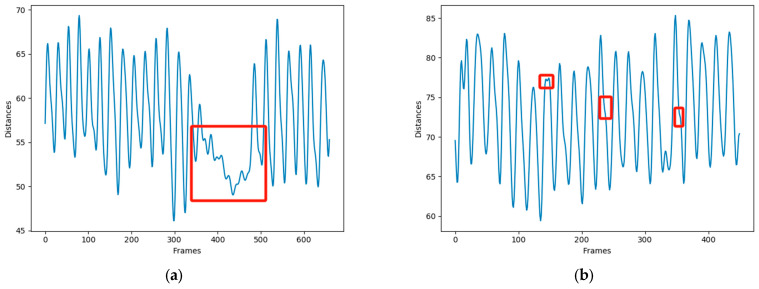
(**a**) Rest behavior. (**b**) Shaking behavior.

Based on this characteristic, we can calculate the number of chews using a peak detection method. However, as seen in Figure 11b, cows may take brief rests during rumination, making it impractical to simply use a method of finding maximum. To address this, we have established a threshold to exclude invalid peaks that do not result from rumination activities. Observations indicate that a single chewing episode for a cow lasts about 20 frames. To reduce errors caused by shaking, it is also necessary to ensure that the frame difference between two consecutive chews, or two adjacent maxima, must be greater than 20. The formula for counting the number of rumination chews is presented in Equation (2).

(2) $$\left\{\begin{array}{c} d_{i}-d_{i-1}>0\\ d_{i}-d_{i+1}>0\\ d_{i}>threshold\\ peak\_index[i]-peak\_index[i-1]>20 \end{array}\right.$$

Due to varying shooting distances, the distances between keypoints also differ. Additionally, during the experiment, it was observed that ideally, counting the number of peaks would suffice to determine the number of chews. However, this is not feasible in practical scenarios because cows occasionally take brief rests during rumination, and the peaks generated during these rests cannot be considered indicative of chewing, as seen in Figure 11a.

Therefore, using a fixed threshold is impractical. We adopted a dynamic threshold approach, specifically setting thresholds based on different scenarios. In this study, the median of the distances between keypoints is used as the threshold. The choice of the median is due to its resilience against outliers. Furthermore, our analysis of numerous instances of rest during rumination showed that fluctuations during these rest periods do not exceed the median value.

To provide a clearer understanding of the implementation process of this method, we have made a brief summary. First, the coordinates of the cow’s nose and mouth are obtained through a keypoint detection model, and the Euclidean distance between them is calculated to generate the rumination motion curve. Then, we smoothed the curve using a low-pass filter to remove noise. Finally, we employed a peak detection algorithm to count the effective chewing actions. Additionally, dynamic thresholds were implemented to eliminate errors caused by resting behavior, and a minimum interval of 20 frames between two chewing actions was set to exclude errors caused by swinging movements.

### 2.7. Evaluation Metrics

#### 2.7.1. Keypoint Detection Model Evaluation Metrics

In target detection tasks, intersection over union (IOU) is used as a measure of similarity, defining the degree of match between the actual target and the predicted target. Thus, the core idea of the measurement method in keypoint detection tasks is to refer to the measurement methods used in target detection tasks. Currently, the evaluation metric for keypoint detection algorithms is object keypoint similarity (*OKS*). Formula (3) is the calculation formula for *OKS*.

(3) $$OKS=\frac{\sum_{i}\left[exp\left(-d_{i}^{2}/2s_{p}^{2}\sigma_{i}^{2}\right)\cdot \delta (v_{i}>0)\right]}{\sum_{i}\left[\delta \left(v_{i}>0\right)\right]}$$

where $d_{i}$ is the Euclidean distance between the predicted position and the actual position of the *i*th keypoint; $s_{p}$ is the area of the target bounding box; $\sigma_{i}$ is the standard deviation between the annotated and actual values of the *i*th keypoint; and $v_{i}$ is the visibility of the *i*th keypoint.

Thus, we calculate the average precision (*AP*) using *OKS* to assess the accuracy of the model. We set a threshold T for *AP*; if the *OKS* exceeds T, it indicates successful keypoint detection. Specifically, *AP_@0.5_* refers to the *AP* value when the *OKS* threshold is 0.5. *AP_@0.5:0.95_* indicates the *AP* values when the *OKS* threshold ranges from 0.50 to 0.95, with increments of 0.05. The *AP* was calculated using Formula (4). Mean average precision (*mAP*) is the average of the average precision (*AP*) values across all categories. From this *AP*, we calculate the *mAP*, which serves as the evaluation metric for keypoints. The *mAP* was calculated using Formula (5).

(4) $$AP=\frac{\sum_{m}\sum_{p}OKS}{\sum_{m}\sum_{p}1}\left(OKS=\left\{\begin{array}{c} OKS(OKS>T)\\ 0(OKS\leq T) \end{array}\right.\right)$$

(5) $$mAP=\frac{\sum_{i=1}^{N}{AP}_{i}}{N}$$

Additionally, precision and recall are common evaluation metrics used in target detection tasks. Precision represents the proportion of correct predictions among the predicted keypoints, while recall indicates the proportion of correct predictions out of all the actual keypoints. Specifically, true positives (TP) represent the number of samples correctly predicted as positive by the model, false positives (FP) denote the number of negative samples incorrectly predicted as positive, and false negatives (FN) represent the number of positive samples incorrectly predicted as negative. Formula (6) is the calculation formula for precision, and Formula (7) is the calculation formula for recall.

(6) $$Precision=\frac{TP}{\left(TP+TN\right)}$$

(7) $$Recall=\frac{TP}{\left(TP+FN\right)}$$

Finally, from an engineering perspective, this study selects parameters and GFLOPs as the evaluation criteria for measuring the size of the model.

#### 2.7.2. Chewing Count Evaluation Metrics

The error rate (ER, %) for rumination chewing is calculated by taking the absolute difference between the predicted number of chews $\hat{y}$ and the actual number of chews y, divided by the actual number of chews. The formula to calculate the error rate of rumination chewing is shown as Equation (8).

(8) $$ER=\frac{\left|\hat{y}-y\right|}{y}\times 100\%$$

## 3. Results

### 3.1. Improved Results for YOLOv8-Pose

#### 3.1.1. Comparison between YOLO-Pose Versions

To more accurately obtain parameters related to cow rumination, we compared the YOLOv5-pose, YOLOv7-pose, and YOLOv8-pose models and the improved YOLOv8-pose in keypoint detection. Table 1 shows the training effects of different models, revealing that both in terms of mAP and model size, YOLOv8-pose overall outperforms YOLOv5-pose and YOLOv7-pose. This demonstrates that improvements based on the YOLOv8-pose network model are feasible.

#### 3.1.2. Ablation Experiment

To assess the impact of adding the GPFN module, ECA attention mechanism, and SimSPPF module on the recognition accuracy of YOLOv8-pose, we conducted ablation studies, with the results shown in Table 2. After adding the GPFN module to the feature fusion section, precision improved by 1.2%, recall improved by 2.2%, and mAP improved by 1.5%, with an increase of 2.2 MB in model size. Introducing the ECA attention mechanism into the feature extraction network resulted in a 3.9% increase in precision, a 2.4% increase in recall, a 1.3% increase in mAP, and no change in model size. Replacing the feature extraction network with the SimSPPF module led to a 2% increase in precision, a 0.5% increase in recall, and a 1.6% increase in mAP, without any change in model size.

Subsequently, we combined these modules in various arrangements within the network, resulting in improvements in precision, recall, and mAP. Finally, by adding all three modules to the network simultaneously, precision increased by 4.5%, recall increased by 4.2%, and mAP increased by 2.8%, with an increase of 2.4 MB in model size. Compared to the original YOLOv8-pose network model, the improved YOLOv8-pose demonstrated significant enhancements in precision, recall, and mAP, laying a solid foundation for the further recognition of cow rumination behavior.

#### 3.1.3. Keypoint Detection Results

**Figure 12:**
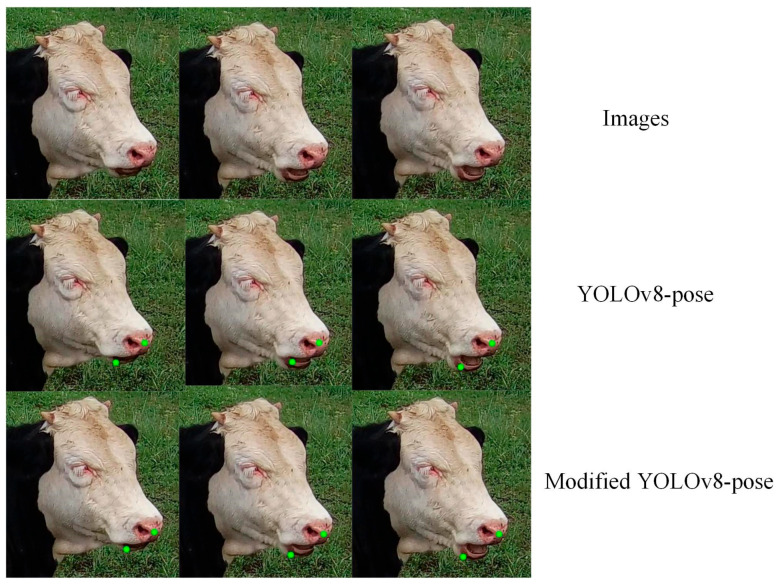
Keypoint prediction results.

We selected a complete cycle of chewing for evaluation, which specifically includes the mouth being closed, slightly open, and fully open. It was observed that both YOLOv8-pose and the improved YOLOv8-pose have commendable keypoint detection outcomes. However, the improved YOLOv8-pose demonstrates more accurate detection results compared to the original YOLOv8-pose, with predicted positions that are closer to the actual annotated locations. Figure 12 displays the keypoint predictions using both YOLOv8-pose and the improved YOLOv8-pose.

### 3.2. Results of Ruminating Chew Count

To validate the feasibility of the chosen threshold, we selected several video segments for testing and compared them with manual observations. As indicated in Table 3, using the median as the threshold for determining rumination is viable, with an average error in rumination chew counts of 5.6% and a standard error of 2.23%. Notably, as video length increased, some videos showed higher error rates due to cows turning their heads during rumination, resulting in the cameras failing to capture the heads and the model missing keypoint information. We will propose improvements in the discussion section of our study. Overall, our model demonstrates high accuracy and reliability in monitoring cattle rumination behavior.

### 3.3. Model Applications

**Figure 13:**
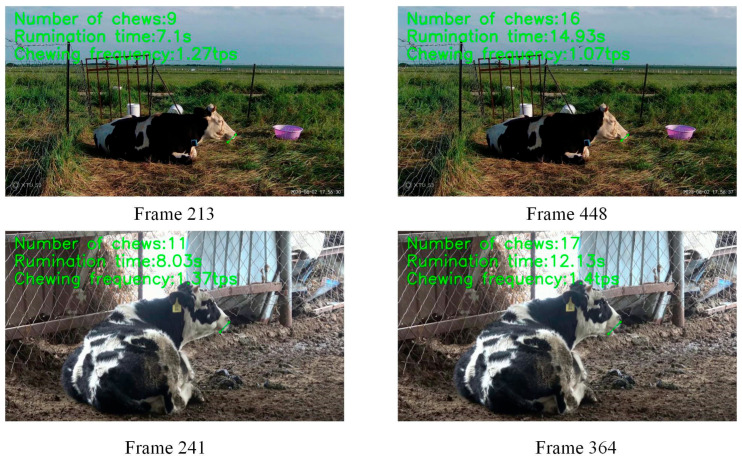
Rumination behavior analysis framework.

In the field of agricultural breeding, the rumination behavior of cattle is of significant importance to their health and productive performance. Thus, implementing the automated monitoring and analysis of cattle’s rumination behavior is a key element in enhancing farm management efficiency and ensuring the health of cattle. This study is dedicated to designing a comprehensive cattle rumination monitoring framework that enables thorough monitoring and in-depth analysis of bovine rumination behavior. This framework includes the following three key indicators: the number of chews, rumination duration, and real-time chewing frequency, which together provide a comprehensive understanding of the cattle’s rumination status. Additionally, we plan to develop an interactive platform to provide breeders with intuitive and convenient tools for data visualization and analysis. Through this platform, breeders can monitor cattle rumination behavior anytime and anywhere, making timely management adjustments to improve breeding efficiency and cattle health levels. Figure 13 illustrates three rumination indicators, with rumination chew count, rumination duration, and rumination frequency displayed from top to bottom. The rumination chew count represents the number of chewing actions during rumination, counting once from mouth opening to closing. Rumination time indicates the time spent on rumination behavior. Notably, if a cow takes a brief rest during rumination, we have set a condition in the framework that the rest time exceeding three seconds is not included in the rumination duration statistics. Chewing frequency represents the ratio of rumination chew counts to rumination duration.

## 4. Discussion

The discussion section primarily validates the robustness and practicality of the model, and it delves into factors that might affect the accuracy of the model’s measurements. We have studied factors influencing the results of keypoint detection and rumination behavior recognition, as well as improvements to the model. Additionally, we analyzed the rumination data in relation to the cows’ health and discussed the potential future applications of this model. We will discuss the limitations of our framework in the following aspects, along with potential solutions.

### 4.1. Impact of Different Modules on Model Improvements

In terms of model improvement, we first compared different keypoint detection models and ultimately chose to enhance the YOLOv8-pose model because it outperformed other models. As shown in Tabel 1, both YOLOv5-pose and YOLOv7-pose have relatively high model parameters and computational complexity. Even with pretrained weights, their accuracy in predicting keypoints was still inferior to YOLOv8-pose. Yuan et al. [28] introduced an improved YOLOv8-ACU network model specifically designed for the identification of facial acupoints, which, compared to the original YOLOv8-pose model, achieved a 1.3% increase in mAP and a reduction in model size by 0.82MB, thereby enabling the precise detection of acupoint locations. Wang et al. [29] developed a single-stage YOLOv8-sp pose estimation algorithm to detect changes in human posture during movement, enhancing the model’s overall accuracy from 73.2% to 89% and providing a reliable tool for analyzing athletes’ joint angles. Qu et al. [30] proposed an improved YOLOv8-pose network model for recognizing pen-holding gestures. Additionally, we experimented with adding various modules to increase the model’s detection accuracy, ultimately achieving a 2.8% improvement in mAP over the original model, as shown in Table 2. Firstly, we replaced the feature extraction network with the SimSPPF module to reduce the model’s computational complexity and accelerate its convergence. Next, we incorporated the ECA attention mechanism, which enhances important features while suppressing irrelevant ones by weighting different channels. This improved feature representation and overall model performance without significantly increasing computational load. Finally, we restructured the model’s neck using the RepGFPN network. This improved the feature pyramid network’s effectiveness and reduced the model’s complexity, thereby enhancing efficiency while maintaining performance.

In addition, there have been some research advancements in the monitoring of rumination behavior. Reiter et al. [31] developed the Smartwell video analysis system, which uses the Smartbow algorithm to monitor the hourly rumination time of dairy cows. Wearable devices for monitoring cow rumination behavior based on multi-source information sensing can track chewing acceleration, sound signals, and posture information [32,33]. While this technology offers high accuracy and real-time monitoring, it comes with high installation and equipment costs and can cause stress responses in cows. Overall, the rumination behavior monitoring method proposed in this study has high accuracy and, being non-contact, effectively avoids stress issues in cows.

In the future, we plan to further refine our model while ensuring accuracy by utilizing pruning techniques or implementing lighter modules to reduce computational demands and compress the model size. This will enable our model to detect keypoint information more rapidly and adapt to real-time device deployment challenges.

### 4.2. Impact of Turning Head on Keypoint Detection

**Figure 14:**
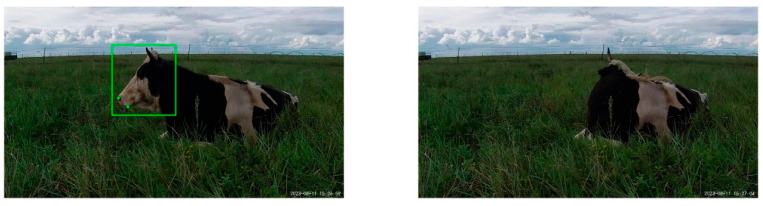
Head-turning behavior.

During the filming process, we used various angles, including front, left, right, and rear side views, to help generalize the model. However, as shown in Figure 14, the model struggles to detect the keypoints of the cow when it turns its head away from the camera, failing to identify the cow’s head and its corresponding keypoints. To address this issue, we plan to install multiple cameras at different positions to cover a broader range of angles and perspectives. Specifically, cameras are arranged at the front, left, and right sides. First, time synchronization is performed for all cameras. The improved YOLOv8-pose model is independently run on each camera to detect the keypoints of the cows, recording the timestamps and the coordinates of the keypoints. Then, based on the timestamps, the keypoint information captured by each camera at the same moment is processed, selecting the most reliable keypoints through confidence screening. Finally, the keypoint information from each camera is integrated into a time sequence to generate a complete rumination behavior motion curve. By implementing this approach, even if the cow turns its head away from one camera, the other cameras will still be able to capture the essential keypoint information of the cow.

### 4.3. Impact of Multiple Targets on Monitoring Rumination Behavior

**Figure 15:**
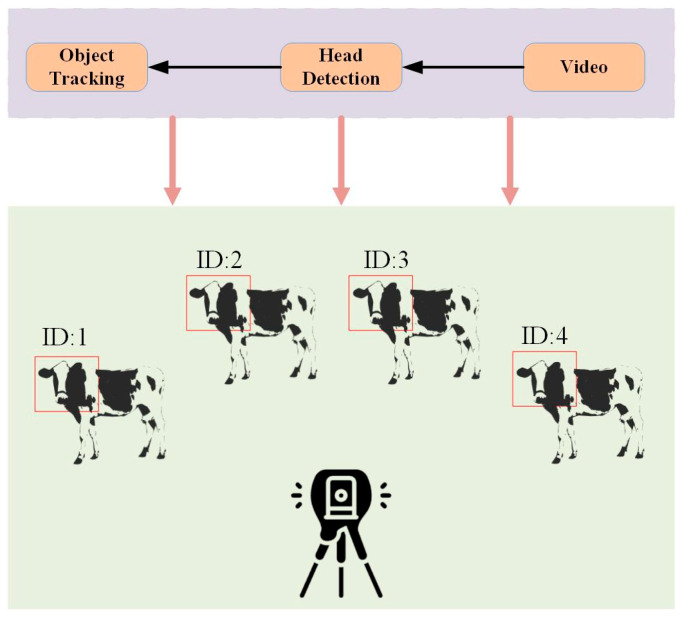
Flowchart of the method.

During the experiment, since the cows were free to move around, it was common for two or more cows to appear in the video simultaneously. This posed significant challenges in monitoring the rumination behavior of a single cow because the keypoint detection process would also capture other cows in the frame. Consequently, this increased the complexity of analyzing the rumination behavior of individual cows. Therefore, we plan to employ multi-object tracking techniques in the later stages of our study to distinguish the keypoint information of each cow [34,35], allowing for the monitoring and analysis of rumination behavior specific to individual animals. Specifically, a target tracking algorithm is employed in the process of cow head detection, allowing each cow to be assigned a unique ID when detecting the herd in the video. The keypoint information is then analyzed based on each cow’s ID, enabling the monitoring of the rumination behavior of each individual cow. Figure 15 provides a flowchart of this method. Even in complex environments in which herds of cows move freely, this approach will enable us to precisely monitor and analyze the rumination behavior of the entire herd.

### 4.4. The Impact of Rumination Behavior on Cattle Health

This study proposes an automated framework for analyzing rumination behavior in cattle, capable of monitoring chewing counts, rumination time, and chewing frequency. These indicators are significantly correlated with cattle health. By observing changes in chewing frequency and rumination time, it is possible to assess the cattle’s feed intake condition [36]. Heinrichs et al. [37] evaluated the rumination time of dairy cows with restricted total mixed ration (TMR) supply, finding that rumination time significantly increased during TMR restriction, regardless of whether hay was provided. Schirmann et al. [38] studied the feeding and rumination behavior of 42 Holstein cows during the early dry period and observed that cows ruminated longer after periods of high feed intake. Understanding cattle rumination patterns can help adjust feed nutrient ratios and dry matter intake, optimize feeding strategies, promote healthy growth, and increase farm profitability. Moreover, the duration of rumination is closely related to cattle production. Cavallini et al. [39] conducted a four-week experiment on 24 Holstein cows and found that reduced rumination time significantly decreased milk production. Antanaitis et al. [40] utilized RuniWatch sensors to monitor changes in rumination behavior parameters before, during, and after calving, allowing for the identification of cows at high risk of subclinical ketosis. Moreover, chewing increases saliva secretion, which helps reduce the risk of acidosis [41]. These rumination indicators can serve as important metrics for veterinary assessments, allowing for a quick understanding of recovery status, reducing treatment costs, and improving treatment efficiency. In summary, the automated framework proposed in this study enables a faster understanding of cattle rumination activities, assisting managers in making timely decisions and achieving intelligent and digital farm management.

### 4.5. Future Application Prospects

This study proposes a non-contact method for monitoring cattle rumination behavior, aiming to enhance livestock management efficiency and reduce costs through advanced technology, aligning with the development direction of smart agriculture. The automated framework proposed in this study can monitor cattle health status in real time, helping livestock managers quickly and accurately identify health issues during the decision-making process, thus enabling timely intervention measures. The specific implementation steps are as follows:

(1) Collect cattle rumination behavior data through cameras;
(2) Transmit the collected data to a server, where model inference is performed to output cattle rumination activity indicators;
(3) Push these indicators to the client side, allowing farm personnel to view this information at any time;
(4) Make scientific management decisions by combining the professional experience of veterinarians.

This method offers significant advantages in improving precision management in livestock farming. It not only reduces the time and labor costs associated with manual monitoring but also minimizes stress responses in cattle and optimizes management processes. In the future, we will continue to study more cattle breeds and optimize the model to adapt to actual farming scenarios, further enhancing the intelligence and efficiency of livestock management. Education and training are crucial in promoting innovative methods [42,43]. By introducing advanced technologies and best practices, future veterinarians and technicians will be better equipped to tackle various challenges in agricultural production, driving livestock management toward a more intelligent and efficient direction.

## 5. Conclusions

This study presents a cattle rumination monitoring framework that employs keypoint detection and applies multi-threshold constraints to filter out interfering behaviors, followed by peak detection to accurately count the number of chews. The main conclusions were as follows:

(1) We enhanced the YOLOv8-pose model by adding three modules, including replacing the feature extraction network with the SimSPPF module and incorporating the ECA attention mechanism with the RepGPFN module used in the neck. The improved YOLOv8-pose achieved a mAP of 96% and an increase of 2.8% and accurately captured relevant keypoint coordinates;
(2) In the rumination module, the average error in chewing count was 5.6%, with a standard error of 2.23% and an accuracy of 94.4%;
(3) A comprehensive cow rumination detection framework was designed to track various rumination indicators, including chewing count, rumination time, and chewing frequency. This framework helps livestock managers observe cow rumination behavior. Understanding changes in rumination behavior during illness can provide more information for the early diagnosis and monitoring of diseases.

However, this study also has certain limitations. First, the variety of cattle involved in this study is too limited; we plan to investigate more breeds in the future to address this limitation. Secondly, during the experiment, we encountered issues with a weak signal at the site, which is not conducive to the real-time transmission and processing of data. To resolve this issue, we plan to implement edge computing to reduce the bandwidth requirements for data transmission delays over the network, thereby improving real-time capabilities and response speed. These improvements will make our framework more practical and promote the sustainable development of precision agriculture.

## Figures and Tables

**Table 1:** Model evaluation results for different versions.

Model	Parameters (M)	GFLOPs (G)	mAP (%)	Model Size (MB)
YOLOv5-pose	33.82	51.3	83.6	68.1
YOLOv7-pose	76.19	101.5	86	153.4
YOLOv8-pose	10.89	29.6	93.2	22
Modified YOLOv8-pose	11.83	30.2	96	24.2

**Table 2:** Results of ablation experiments.

YOLOv8-Pose	RepGPFN	ECA	SimSPPF	Precision (%)	Recall (%)	mAp (%)	Model Size (MB)
√				92.3	83	93.2	22
√	√			93.5	85.5	94.7	24.2
√		√		96.2	87.1	94.5	22
√			√	95.3	88	94.8	22
√	√	√		94.9	86.2	95.3	24.3
√	√		√	96.4	88.1	95.1	24.2
√		√	√	95.4	87.6	95.4	22
√	√	√	√	96.8	87.2	96	24.2

**Table 3:** Chewing count statistics results.

Number	Video Time Length/(s)	Assessed Value/(Times)	Measured Value/(Times)	Relative Error/(%)
1	74	83	79	4.82
2	108	122	119	2.46
3	164	130	120	7.69
4	231	267	277	3.75
5	248	291	283	2.74
6	300	393	376	4.33
7	259	340	321	5.59
8	348	379	345	8.97
9	256	214	198	7.47
10	242	280	257	8.21
Mean				5.6
Standard error				2.23

## Data Availability

Data are available on request from the authors.
